# Buffalo Law School

**Published:** 1887-10

**Authors:** 


### *BUFFALO LAW SCHOOL—LAW DEPARTMENT, NIAGARA UNIVERSITY.*

The opening of the Buffalo Law School occurs on Monday, October 3d. All students are requested to report on Saturday, October 1st, at ten o'clock A. M., at the Law School lecture-room, in the building of the Medical Department of the Niagara University, on Ellicott street, opposite the Buffalo Library, for the purpose of examination and registering.

The formal opening of the school will be held on Monday evening, October 3d, at the lecture-room in the Buffalo Library, at eight o'clock. Hon. Charles Daniels, LL.D., dean of the law faculty, will deliver the principal address. Citizens, generally, are invited to be present. The hours of instruction will be in the morning of each day, from nine to eleven o'clock. Moot courts will be held on Saturday mornings. The course of instruction will be substantially as follows:

FOR THE JUNIOR CLASS.

Contracts and Private Rights — Leroy Parker, three hours each week for the entire year: Tuesdays, ten to eleven, Wednesdays, nine to ten, Thursdays, ten to eleven.

Practice in Civil Actions — Charles P. Norton, three hours each week for the entire year: Mondays, ten to eleven, Wednesdays, ten to eleven, Fridays, nine to ten.

Property — Spencer Clinton, Tuesdays, from nine to ten, fifteen weeks. Mr. Clinton will be supplemented by a course on real estate. E. L. Parker, Tuesdays from nine to ten, fifteen weeks.

Theory of the Codes and Codifications — John G. Milburn, Mondays, nine to ten, ten weeks.

Criminal Law and Practice — Tracy C. Becker, Mondays, nine to ten, fifteen weeks.

Torts—Hon. George S. Wardwell, Thursdays, nine to ten, twenty weeks.

Law of Corporations — Charles B. Wheeler, Fridays, ten to eleven, ten weeks.

Wills and Estates of Deceased Persons — Hon. Jacob Stern, one hour each week: Thursdays, nine to ten, ten weeks.

Marriage and Divorce — Lecturer to be hereafter announced, one hour each week: Fridays ten to eleven, twenty weeks.

SENIOR YEAR.

Evidence — Adelbert Moot, one hour each week; Mondays, nine to ten, twenty weeks.

Equity Jurisdiction—Hon. Charles Beckwith, one hour each week: Wednesdays, nine to ten, fifteen weeks.

Mercantile Law—Leroy Parker, two hours eack week for the entire year: Mondays, ten to eleven, Fridays, nine to ten.

Corporations — James Frazer Gluck, one hour each week: Wednesdays, ten to eleven, fifteen weeks.

Medical Jurisprudence—Tracy C. Becker, one hour each week: Mondays, ten to eleven, fifteen weeks.

Maritime Law — Hon. George Clinton, one hour a week: Thursdays, ten to eleven, fifteen weeks.

Constitutional Law—Hon. Charles Daniels, one hour each week: Thursdays, ten to eleven, fifteen weeks.

Legal Ethics — Hon. Albion W. Tourgee, six lectures; dates will be announced.

Trials—Hon. L. L. Lewis, one hour each week: Wednesdays, ten to eleven, ten weeks.

Taxation — Lecture to be announced, one hour a week: Wednesdays, ten to eleven, ten weeks.

Trusts — Hon. L. N. Bangs, once a week: Fridays, ten to eleven, twenty weeks.

Roman Civil Law — Sheldon T. Viele, once a week: Tuesdays, nine to ten, ten weeks.

International Law—Hon. S. S. Rogers, once a week: Wednesdays, ten to eleven, ten weeks.

Practice in Civil Actions — Charles P. Norton, two hours each week: Tuesdays, ten to eleven, Thursdays, nine to ten, for the entire year.

